# Arteriovenous Malformation With Intranidal Giant Aneurysm: A Case Report

**DOI:** 10.7759/cureus.89703

**Published:** 2025-08-09

**Authors:** Gilbert Paul B Distura, Reynaldo Benedict V Villamor

**Affiliations:** 1 Department of Neurosurgery, Vicente Sotto Memorial Medical Center, Cebu, PHL

**Keywords:** arteriovenous malformation, avm surgery, giant aneurysm, intranidal aneurysm, neurosurgery

## Abstract

The coexistence of intracranial aneurysms and brain arteriovenous malformations (AVMs) and their relationship presents significant diagnostic and treatment difficulties. The existence of an intranidal aneurysm elevates the risk of bleeding and complicates the management strategy. A 45-year-old man arrived with weakness on the right side, difficulty speaking, facial weakness, and a severe headache. This case highlights the significance of timely identification and prompt surgical intervention for giant aneurysms linked with AVMs to avert potentially dangerous hemorrhagic occurrences and enhance patient outcomes.

## Introduction

Arteriovenous malformations (AVMs) are a vascular developmental disorder characterized by clusters of inadequately formed blood vessels where the supplying arteries connect directly to a venous drainage system without any intervening capillary network [1-4]. Brain AVMs are of particular concern due to the elevated risk of hemorrhage from the abnormal blood vessels, which can lead to central nervous system injury. Brain arteriovenous malformations significantly contribute to intracranial hemorrhage, particularly in younger people, and are linked to higher rates of morbidity and mortality.

The occurrence of aneurysms in individuals with AVMs is greater than anticipated when considering the prevalence of each condition separately. They are regarded as uncommon and appear in fewer than 1% of the overall population. They frequently occur in younger adults, with a morbidity rate of 30-50% and a 10-15% risk of mortality. Individuals with cerebral arteriovenous malformations face a higher risk of bleeding when an intranidal aneurysm, an aneurysm located within the core (nidus) of the malformation, is present [5].

Intranidal aneurysms are often linked with AVMs, likely because of the hemodynamic pressures applied to the arterial wall. The occurrence of such a link has been documented to range from 5.5% to 12%. The majority of these aneurysms are small or medium-sized, whereas giant aneurysms are extremely uncommon, with only a handful of cases reported [6].

Information regarding AVMs in the Philippines is insufficient. Over the past two decades, only four journal articles [7-10] have been published regarding AVMs, each involving a limited number of Filipino patients: a 10-year-old female with a giant intracranial aneurysm [7], an 89-year-old Filipino female with a ruptured middle cerebral artery aneurysm within a supratentorial ependymoma [8], a retrospective cohort of adult patients with aneurysmal subarachnoid hemorrhage treated at a major public hospital [9], and a series of pediatric intracranial aneurysm cases managed at a tertiary center [10].

The most recent study was conducted by Gigataras and Legaspi in 2006 [11], which offered information on the management of these lesions through a prolonged series at a single institution in the Philippines. Moreover, publications on giant intranidal aneurysms are infrequently addressed and released. Consequently, this case study merits reporting and publication to enhance the current understanding of intranidal giant aneurysms.

## Case presentation

A 45-year-old married man presented with weakness on the right side, difficulty speaking, uneven facial features on the left, and an intense headache. The motor weakness was limited to the face, as limb strength was found to be normal on neurological examination. Five days before admission, he had a brief episode of loss of consciousness accompanied by vomiting. Upon hospital admission, the patient experienced intermittent confusion and persistent frontal headaches. His medical history revealed a previously diagnosed cerebral aneurysm in 2018, for which no surgical or endovascular intervention had been performed. He denied any history of hypertension, diabetes, or asthma. The patient was a non-smoker, did not use illicit drugs, and consumed alcohol only during special occasions. He was employed as a security guard, and both of his parents had a history of diabetes mellitus.

On physical examination, the patient was awake and oriented to time, place, and person. Vital signs were within normal limits. Neurological assessment showed symmetrical muscle strength and intact deep tendon reflexes in all extremities. However, slurred speech and right-sided facial asymmetry were observed. There were no abnormal reflexes or visual disturbances.

**Figure 1:**
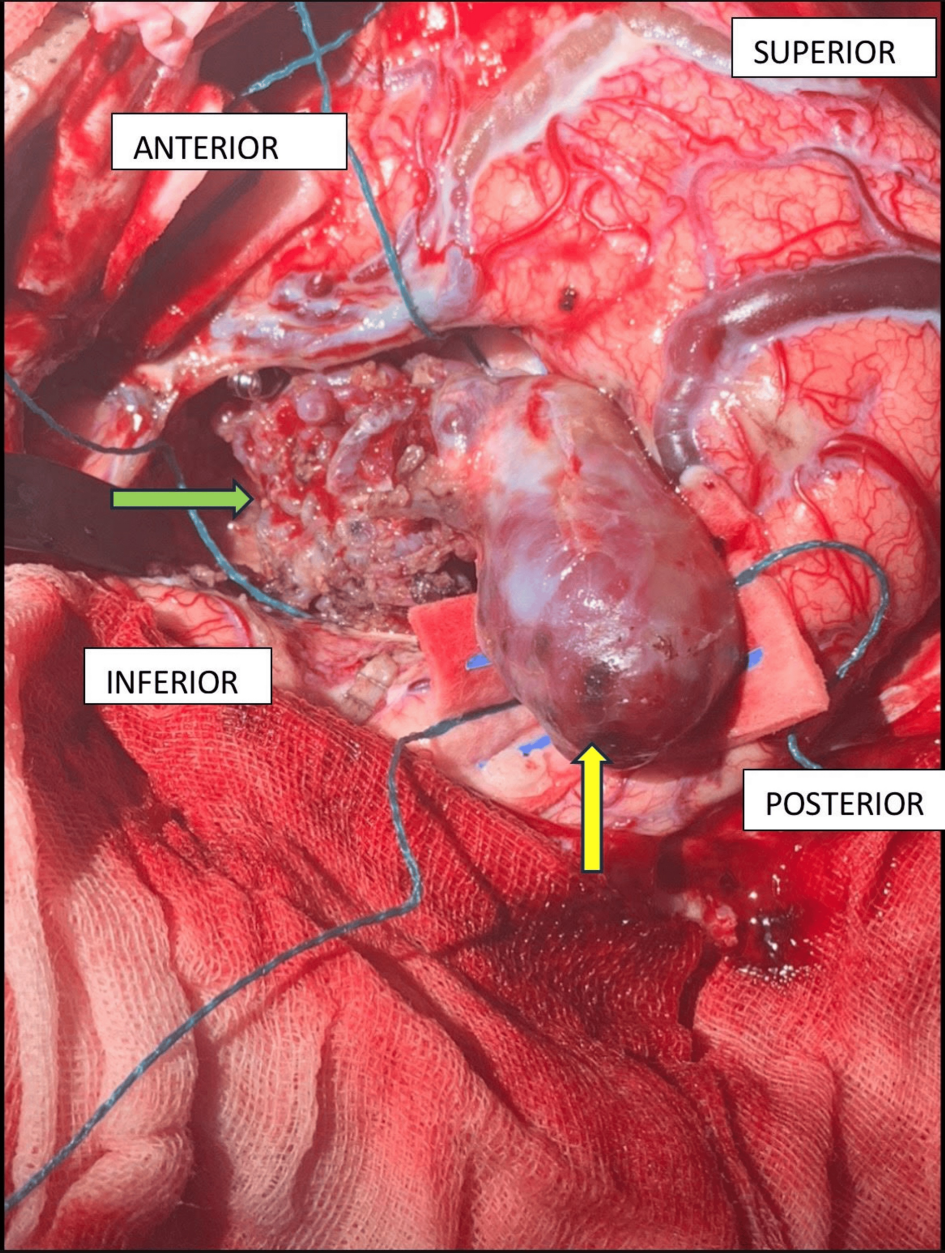
Intraoperative findings Intraoperative findings showing intranidal giant aneurysm (yellow arrow) and arteriovenous malformation (green arrow)

Cranial CT and MRI revealed a significant AVM located in the right temporoparietal region. The arterial supply originated from branches of the left middle cerebral artery, with venous drainage involving cortical veins emptying into the superior sagittal sinus, left transverse sinus, and left sphenoparietal sinus. The AVM nidus measured approximately 3 × 3 × 4.6 cm and featured a giant intranidal aneurysm measuring 4.7 × 5.3 cm at its medial-posterior aspect. These findings were further confirmed by cerebral catheter angiography, which provided a detailed view of the AVM architecture and its feeding and draining vessels (Figure 1).

This was classified as a brain arteriovenous malformation (BAVM) Spetzler-Martin Grade II, which, based on existing literature, is associated with a favorable prognosis and improved six-month outcomes when managed through surgical excision.

**Figure 2:**
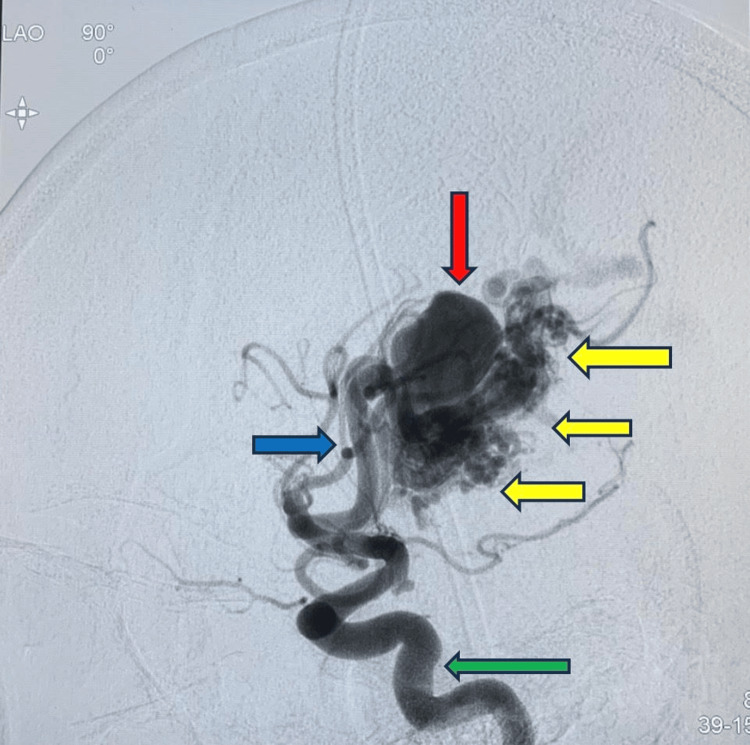
Lateral left ICA injection, early arterial phase Main internal carotid artery (ICA) supply (green arrow); arterial feeder from cortical middle cerebral artery (MCA) branches (blue arrow); arteriovenous malformation with a compact nidus (yellow arrows); and intranidal giant aneurysm (red arrow)

Radiologic imaging was initiated with a cranial CT scan (plain), which revealed subarachnoid hemorrhage with intraparenchymal hematoma, showing a pattern suggestive of a ruptured aneurysm. Based on these findings, a diagnostic cerebral catheter angiography, the gold standard for evaluating vascular malformations, was performed. This confirmed the presence of a significant AVM in the right temporoparietal region. Feeding arteries were selectively ligated, and the draining veins were secured prior to complete excision of the AVM and aneurysm (Figure 2).

**Figure 3:**
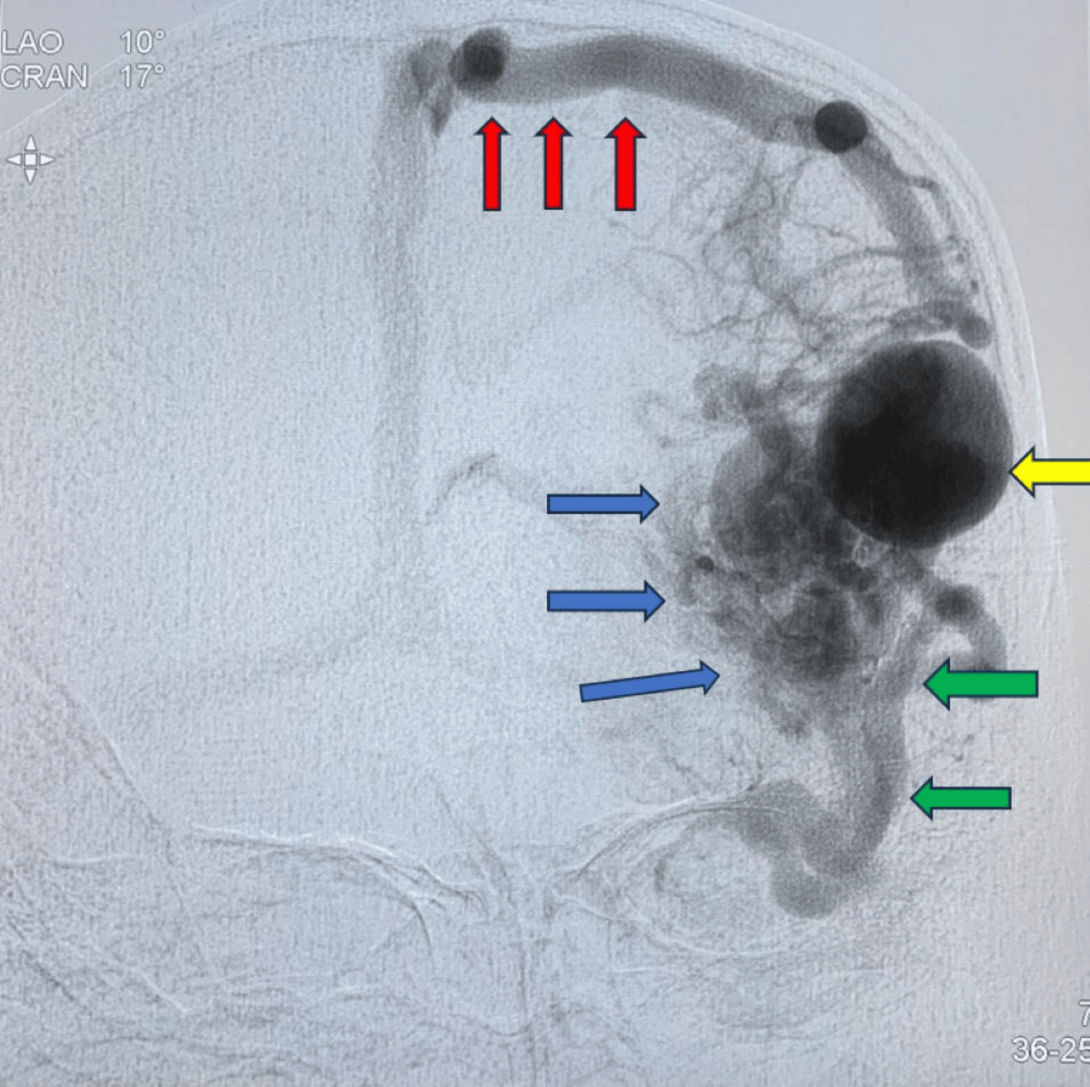
AP view, late arterial phase, left internal carotid artery (ICA) injection. Dilated cortical middle cerebral artery (MCA) branches (green arrows); compact arteriovenous malformation (AVM) (blue arrow); giant intranidal aneurysm (yellow arrow); single draining vein going to the superior sagittal sinus (red arrows)

Figure 3 presents an anteroposterior (AP) view during the late arterial phase following a left internal carotid artery (ICA) injection. The image highlights key vascular abnormalities associated with the AVM. Notably, there is prominent dilation of cortical branches of the middle cerebral artery (MCA) (indicated by green arrows), suggestive of increased arterial flow toward the nidus. A compact AVM nidus is visualized (blue arrow), with a giant intranidal aneurysm seen within the core of the lesion (yellow arrow), representing a high-risk hemorrhagic feature. A single prominent draining vein (red arrows) is directed toward the superior sagittal sinus, indicating a superficial venous drainage pattern. These findings are consistent with a high-flow AVM with intranidal aneurysmal formation and superficial venous outflow.

**Figure 4:**
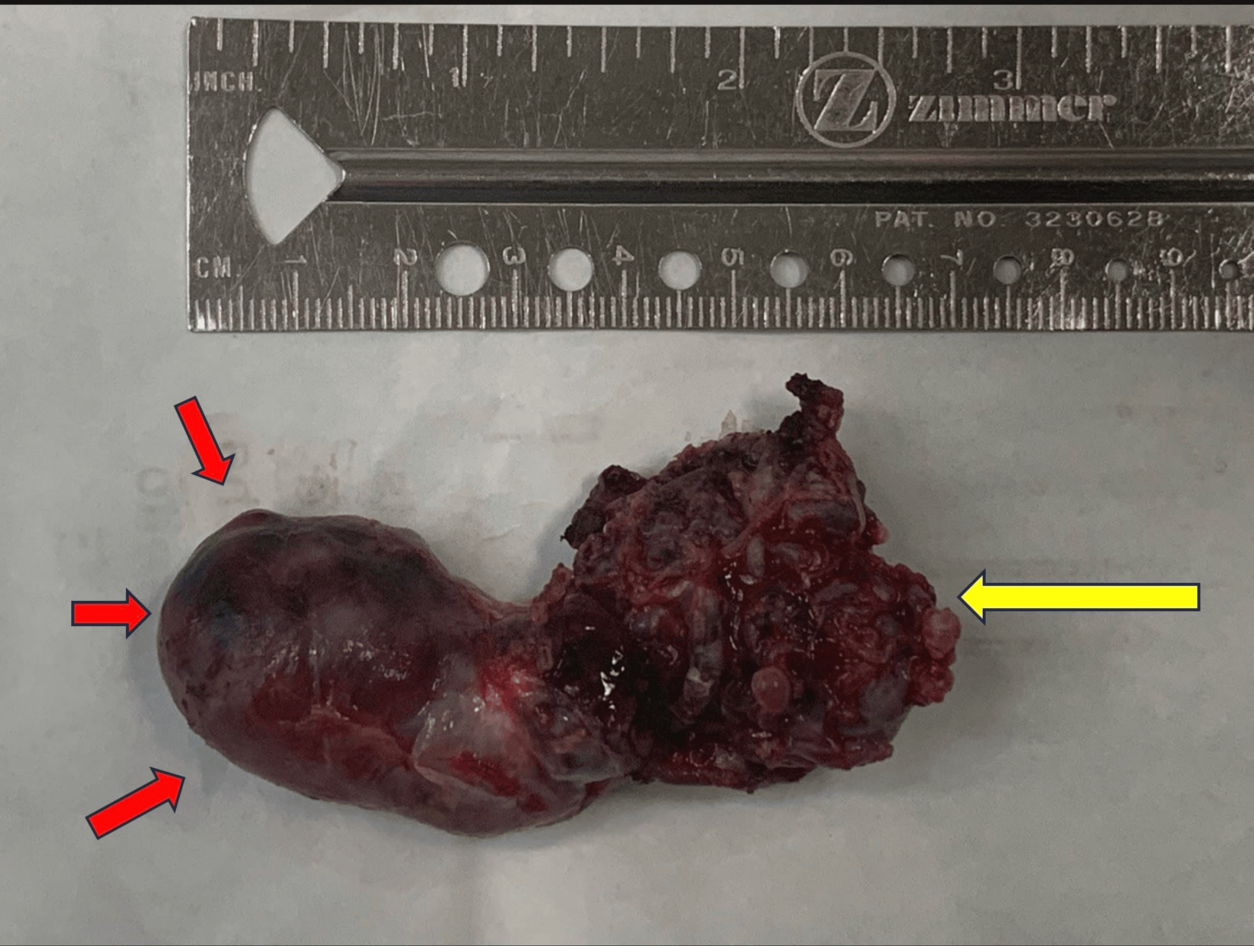
Excised arteriovenous malformation (AVM) Gross specimen showing the compact arteriovenous malformation (yellow arrow) together with the giant intranidal aneurysm (red arrow).

 The resected specimen demonstrated a well-defined AVM with an associated giant aneurysmal component (Figure 4).

**Figure 5:**
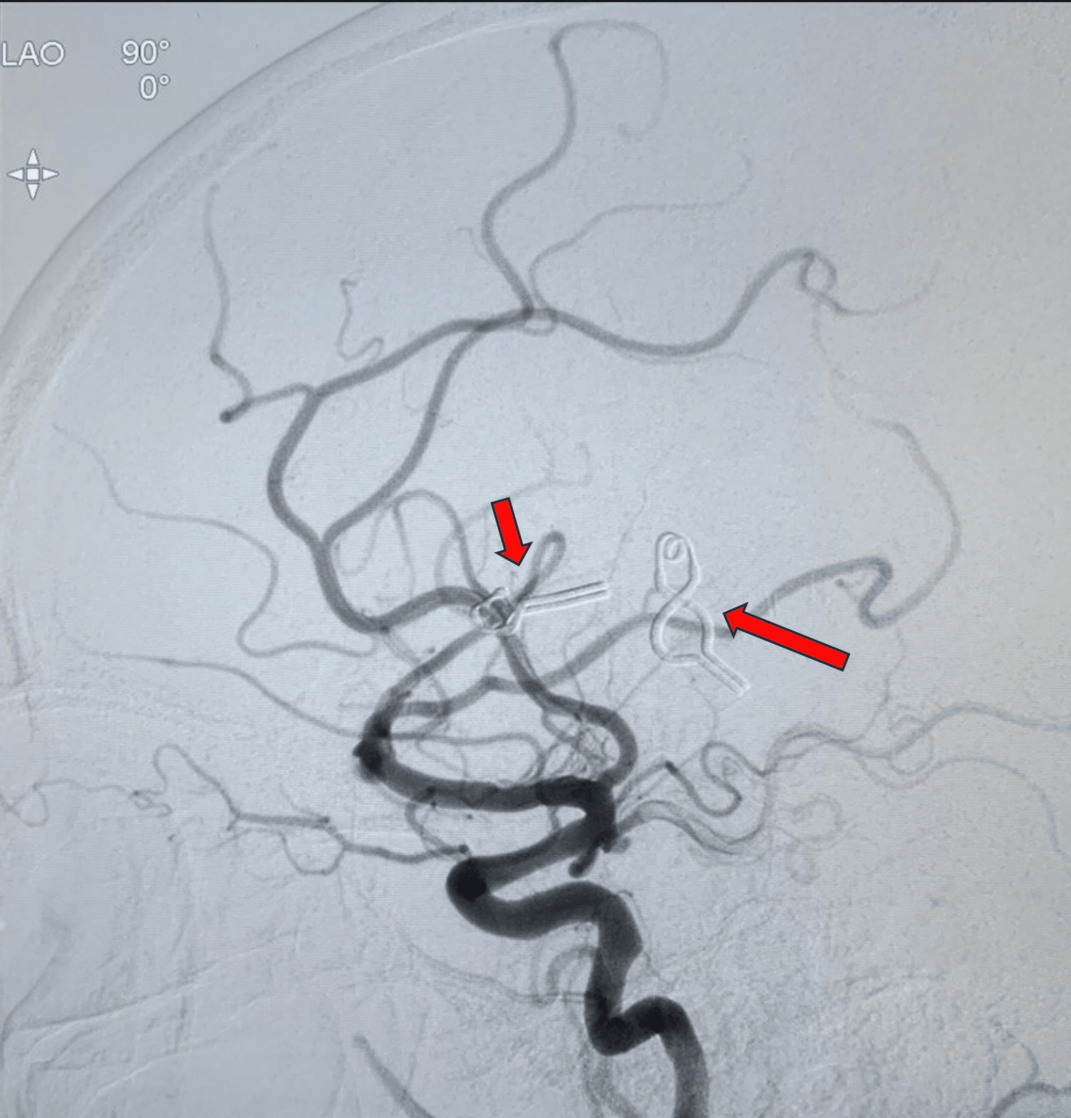
Left internal carotid artery (ICA) injection, post excision Complete excision of the previous compact arteriovenous malformation with giant intranidal aneurysm, with decrease in caliber of previously engorged cortical feeders; multiple clips (red arrows)

Postoperative cerebral catheter angiography confirmed complete resection of the AVM, with no residual shunting or abnormal vascular connections observed (Figure 5).

The patient’s postoperative recovery was uneventful, with no immediate complications. He provided written informed consent for the publication of this case report and its associated images.

## Discussion

AVMs are vascular anomalies defined by a complicated knot of abnormal vessels, in which arteries link directly to veins without the presence of capillaries. This structural irregularity impairs normal blood circulation patterns and heightens the likelihood of bleeding. Hemorrhaging from AVMs with intranidal aneurysms constitutes around 2% of all hemorrhagic strokes, especially impacting young adults [12]. The existence of a related aneurysm, particularly inside the nidus, greatly increases the risk of bleeding. The incidence of aneurysms in patients with AVMs varies depending on the study and classification. For example, the initial collaborative research on intracranial aneurysms revealed that 24% (8 out of 34) of individuals with AVM-associated bleeding had several aneurysms [6]. Similarly, the Scottish AVM study [13] group found 34 aneurysms among 114 patients with unruptured AVMs, though the types of aneurysms were not detailed [13,14]. The individual in this instance falls within the usual age range (11-35 years) deemed at greater risk for bleeding after an AVM diagnosis [15].

Aneurysms linked to AVMs are generally classified according to their site, blood flow characteristics, and tissue structure. These may be either arterial or venous and are additionally categorized as extranidal or intranidal based on their connection to the AVM nidus. Intranidal aneurysms typically fill promptly during angiography and are frequently of venous origin. It is believed that their development is affected by unusual hemodynamic stress resulting from elevated flow shunting within the nidus [16]. Nonetheless, merely a portion of AVM patients experience these aneurysms, indicating that other contributing elements like genetic susceptibility or individual vascular reactions may play a role [6]. Certain research suggests that arterial aneurysms occur more often in older individuals and patients with high-grade AVMs, indicating that they develop over time as a result of prolonged hemodynamic stress. Significantly, unusual AVM hemodynamics have been noted to continue even post-hemorrhage, indicating that bleeding might worsen flow disturbances instead of simply stemming from them [17]. 

There is still no agreement on the best treatment approach for AVMs linked to aneurysms. Surgical removal, endovascular embolization, and radiosurgery are all feasible alternatives, with the decision customized based on the lesion's location, size, rupture condition, and expertise available. In this instance, the AVM containing an intranidal giant aneurysm was effectively managed through open microsurgical resection. Removing the AVM can greatly decrease or remove related aneurysms, especially those found near the nidus. Research indicates that individuals with both AVMs and aneurysms face an increased risk of hemorrhage, 7% in one year compared to 3% for those with AVMs alone. Additionally, the existence of an aneurysm is a standalone predictor of hemorrhage following radiosurgery in low-grade AVMs [18]. Determining the precise origin of the bleeding, whether it is from the AVM or the aneurysm, is essential. If a subarachnoid hemorrhage happens without intraparenchymal bleeding, the aneurysm is typically the probable source and requires urgent treatment. In situations such as the one described, where considerable venous outflow blockage is observed, immediate surgical action is necessary. Recent developments additionally facilitate selective catheterization and embolization of AVM segments containing aneurysms as a new therapeutic option.

## Conclusions

Cerebral AVMs can exist alongside intracranial aneurysms, and their simultaneous occurrence poses a higher risk for hemorrhage compared to AVMs or aneurysms individually. The status of rupture is the key factor in determining the need for immediate treatment, requiring swift action if the aneurysm is found to be the cause of the bleeding. On the other hand, if bleeding arises from the AVM nidus and there is no major obstruction in venous outflow, it is possible to postpone treatment safely. Management approaches for unruptured aneurysms linked to AVMs typically align with those for standalone aneurysms, as certain distal flow-related aneurysms may diminish following AVM intervention. In the end, treatment choices should be personalized according to the lesion's location, type, and clinical features. This case demonstrates the effective surgical removal of a large intranidal aneurysm found within an AVM, leading to a positive clinical result.
